# Distance from a cultural model of substance use risk, internalization, and self-stigma in urban Brazil

**DOI:** 10.3389/fpsyg.2023.1264436

**Published:** 2023-12-18

**Authors:** Nicole L. Henderson, William W. Dressler, Natália Priolli Jora Pegoraro, Ana Falcão, Sandra Cristin Pillon

**Affiliations:** ^1^Division of Hematology and Oncology, Department of Medicine, University of Alabama-Birmingham, Birmingham, AL, United States; ^2^Department of Anthropology, The University of Alabama, Tuscaloosa, AL, United States; ^3^Faculty of Nursing, University of São Paulo-Ribeirão Preto, Ribeirão Preto, Brazil

**Keywords:** cultural models, cultural distance, substance use disorder, self-stigma, Brazil

## Abstract

**Introduction:**

A cognitive theory of culture as socially distributed cultural models has proven useful in research. Cultural models exist in two forms: the model shared by individuals in a social group, and individual versions of that model modified by personal experience. In previous research we documented a shared cultural model of substance use risk among a general population sample in urban Brazil. Here we examine how this model is distributed among persons under treatment for substance use/misuse and the implications for perceived and self-stigma.

**Methods:**

A convenience sample of 133 persons under treatment rated the influence of risk factors for substance use/misuse. The configuration of those ratings and the cultural distance of persons under treatment from the general population model were calculated. Degree of stigma perceived in the wider society and degree of self-stigma were also assessed.

**Results:**

Persons under treatment aggregate risk factors to a greater extent than the general population. Using a cultural distance metric, the more distant persons under treatment are from the general population model, the lower their self-stigma regarding substance use.

**Discussion:**

Some individuals under treatment separate their understanding of substance use/misuse from shared perspectives in the wider society, which in turn reduces self-stigma. These findings add an additional perspective on the relationship of culture and the individual.

## Introduction

One of the enduring theoretical and empirical questions in the social sciences in general, and in anthropology specifically, is the relationship between culture and the individual (Dressler, 2018). Are we merely “cultural dopes” whose thought and action are determined by our culture of upbringing; or, are we independent agents who forge our own paths in life irrespective of cultural influences?

The real empirical question, of course, lies somewhere between these statements. The set of cultural models that inform thought and action certainly shape our understanding of the world; at the same time, individuals incorporate the knowledge encoded in cultural models with their own experience and goals in life. These “subjectivities” in turn guide interpretations of and action in the world (Strauss, 2018a).

Our aim in this paper is to explore these questions in a particular context: cultural influences on stigma in relation to substance use and misuse in urban Brazil. Previous research has shown that there is a shared but relatively nonspecific model of the risk of substance misuse in Brazil that, as a result of drug education and media focus, is centered within a specific demographic: young adults in the general population (Henderson and Dressler, 2020). Here we explore how this model is configured and internalized by persons occupying a different social position: those who are under treatment for substance use. We find that persons under treatment tend to make fewer distinctions among risk factors than do general population young adults. Furthermore, the greater the distance between individuals under treatment and the young adults in terms of the cultural model, the less likely persons under treatment are to self-stigmatize as a result of their substance use. These findings offer a novel perspective on the relationship of culture and the individual.

## Theoretical background

The nature of the relationship between culture and the individual has been a question debated since the beginnings of anthropology. In the early days of the field, Kroeber (1917) thought that individuals should be ignored in favor of the study of culture as an entity external to them, while Sapir (1917) countered that such an external entity was of no theoretical use and questionable empirical status, and that the observed behavior of individuals was where culture was manifest.

Much later both Goodenough and Schwartz attempted to reconcile the external quality of culture with the undeniable fact that persons incorporate culture into their own subjective experience of the world and employ it in their everyday interactions. They posited individual versions of culture [the “propriospect” for Goodenough (1981) and the “idioverse” for Schwartz (1978)] that reflected a combination of culture in the aggregate with personal experience, although neither of these constructs provided much empirical guidance. Spiro’s (1997) concept of “internalization” is also relevant here, in that it describes a step-wise process by which individuals proceed from a mere passing knowledge regarding some cultural domain to actively incorporating that knowledge into a personal belief system that they then use to guide their own actions. Still, an effective way of describing and operationalizing both culture in the aggregate and the subjective experience of culture has eluded researchers.

In part, the ways in which culture is internalized and becomes a part of individual subjectivity depends on how culture itself is conceptualized. Here we rely on a cognitive theory of culture, starting with Goodenough’s (1956) definition of culture as that which one must know in order to act acceptably in a given social group. In contemporary cognitive culture theory, this knowledge is understood to be encoded in the form of cultural models. A cultural model is a schematic outline of some salient cultural domain (itself a focus of discourse within the group) that includes the elements that make up the domain, along with the semantic, functional, and causal relations that are understood to distinguish among and link those elements. Cultural models contain one or more prototypes that can be either some kind of abstract type that represents that domain, or an actual member of the domain thought to represent a best example (D’Andrade, 1995; Bennardo and de Munck, 2013).

Examining cultural models requires that an emic approach be adopted. The term “emic” is derived from linguistics and phonemic analyses, a phoneme being the smallest unit of sound that makes a difference in the meaning of word. An emic approach in ethnography requires that the focus of research is on the meaningful distinctions that members of a community themselves make in understanding the world around them, as opposed to an etic approach in which categories and distinctions are imposed on community members by the researcher (Lett, 1996). An emic approach focuses on “the insider’s view” of specific cultural domains.

Cultural models are most profitably investigated using a mixed-methods approach. In-depth interviewing (including traditional ethnographic interviews, person-centered interviews, and free lists) is essential for eliciting the elements that make up an hypothesized cultural model and for describing how those elements are linked and distinguished (Dengah et al., 2021). Then, a quantitative analysis employing cultural consensus theory (Romney et al., 1986) can be used to verify that the model is shared and hence a cultural model (see Weller et al., 2023). Cultural consensus analysis can also be used to estimate how much of the knowledge encoded in the model is shared within a social group, and it can be used to estimate the most likely content encoded in the model. One of the advantages of the combination of cultural model theory and cultural consensus theory is that culture can be understood, non-mysteriously, as an emergent property of social groups, in that it cannot be reduced to what any given individual knows, but rather is a weighted average of individual knowledge, in which individuals who command more of that knowledge contribute more to the aggregate keeping in mind that, in cultural consensus theory, “to know” something means to agree more with others about it (Dressler, 2018).

Understanding cultural models and individual subjectivities, or how persons incorporate that knowledge into their own thought and action, requires another step. Dressler’s (2018) concept of cultural consonance describes the link between cultural knowledge and individual behavior. Traditionally, this work has focused on the individual’s ability to “live-up” to the expectations encoded in widely shared cultural models and the effects of that “success” or “failure” on measured health outcomes. As Dressler (2018) has argued, there must be an aspirational component to the cultural model that drives one to be culturally consonant. A separate question, however, pertains to how individuals utilize their understandings of shared cultural models to recursively inform and modify their own personal models, or, their subjective experience of the world. This question has been less well investigated.

Strauss has been particularly interested in this question. Her research on economic mobility (Strauss, 1990), political orientations (Strauss, 1997), and poverty (Strauss, 1990) has focused on how individuals take their shared knowledge of cultural models and their own experiences and mold them into ways of thinking about and coping with the world around them. Her approach to this work has emphasized person-centered interviewing and a thick description or interpretive analysis of those narratives to demonstrate how individuals incorporate culture into their daily lives.

This work is rich and illuminates carefully how culture lives in persons. At the same time, the ways that varying subjectivities are socially distributed within the community in question is missing from these analyses [although Strauss (2018b) correctly points out how the description of varying subjectivities described in individual case studies illuminates intracultural variation]. It might be argued that cultural consensus analysis itself achieves this goal by describing variation in cultural competence, the measure of how much knowledge an individual shares with others. But cultural competence does not describe whether or not that knowledge is salient for the individual and incorporated into their experience of the world or, put more simply, whether that knowledge matters individually. In Gatewood’s terms, it may simply be “knowledge of” but not actual “knowledge for” (Gatewood, 2011).

An added complication in conceptualizing the relationship of cultural models and individual experience has been highlighted by Chentsova-Dutton and Ryder (2020): most research on the topic has focused on cultural models that are normalized or valorized within a community. That is, they describe thought and action that is at least understood to be ordinary and perhaps is a life goal for community members (e.g., Dressler et al., 2017). But what of cultural models of domains that are disvalued or encode what is culturally constructed as deviant within that society? One such domain is substance use and misuse. Persons who use or misuse substances are often the objects of stigma. This stigma can take several forms, including attributed and enacted stigma coming from other persons, and/or perceived and self-stigma felt by the persons using substances themselves (Paquette et al., 2018). Perceived and self-stigma, two foci of this paper, can be particularly problematic in that these forms of stigma can inhibit seeking treatment and lead to comorbid mental health problems. Furthermore, recent reviews suggest that understanding of factors underlying these forms of stigma remains limited (Milan and Varescon, 2022).

Here we present research that helps to address these issues. In a study of the stigma associated with substance use and misuse in Brazil, we first documented a cultural model of the risk of substance use among young adult Brazilians in the general population that in turn informed their tendencies to stigmatize—or not—substance users, by labeling them as untrustworthy and dangerous (Henderson and Dressler, 2020). Next, a sample of persons under treatment for substance misuse rated the influence of the same risk factors for substance use based on their own individual experiences. We thus can determine the similarity and differences in cultural models of substance use risk between the general population and those under treatment. This is a particularly interesting example of the relationship between culture and the individual because the cultural model of the general population is, in essence, imposed on the treatment sample in the sense that in everyday interaction they encounter persons who know and/or adhere to that model and expect them to do the same. The proximity or distance of persons under treatment from this model in their own evaluations of risk factors will contribute to an understanding of how cultural models are put to use by individuals, with the added advantage of describing the distribution of models in use.

Furthermore, the idea of “cultural distance” will be employed operationally here, not just metaphorically. There is evidence that in some cultural domains, people experience culture as a space they navigate (Lakoff and Johnson, 1980; Dressler et al., 2023), determining their position in that space relative to prototypes encoded in cultural models. Actually measuring the distance of persons under treatment from a general population model in a multidimensional array will allow us to explore this theoretical orientation further, especially in terms of how this might influence their subjective well-being, as measured by their experience of stigma.

By comparing the treatment sample to the general population sample, we can examine the following research questions:

- how proximate are persons in the treatment sample in their thinking to the general population sample?
- how are the elements of the cultural model of substance use risk reconfigured by persons sharing the status of substance user?; and,
- what are the implications of proximity to or distance from the general population sample for stigma experienced by the substance user?

## Ethnographic setting

Research was conducted in the city of Ribeirão Preto, a community of over 700,000 persons in the north of the state of São Paulo. It sits in a rich agricultural region originally devoted to coffee production and more recently emphasizing sugar cane and citrus. The city itself has become a regional center in manufacturing, finance, and education.

Many sources identify Brazil as a leading consumer of drugs (INPAD, 2012). Approximately 50% of the population engages in recreational alcohol use, 2–3% in cannabis use, and 1–2% in cocaine/crack use (CICAD, 2019). Despite seemingly low prevalence rates, Brazil is the second largest consumer of cocaine in the world (CICAD). Nearly 4% of the adult Brazilian population experiments with cocaine at some point in their lives, and of those nearly half (48%) become dependent on the substance (Pillon et al., 2017). Substance use is particularly popular among young adults and those attending university (Andrade et al., 2010). Houvèssou et al. (2021) found that 92% of undergraduate students surveyed in southern Brazil consumed alcohol, while 13% of students combined alcohol use with the use illicit substances.

The rates of substance use in Ribeirão Preto are also estimated to be relatively high (de Freitas and de Moraes, 2011). Alcohol use is common, fueled in part by the historic beer industry of the city (arguably one of the most famous bars in all Brazil serving *chopp* or draft beer is located there). Cannabis use is common, especially within the large university student population of the city, and there are several well-known local scenes for drug use where crack cocaine users convene to buy and consume the drug (LECUCA, 2020).

While there have been attempts to move public policy in Brazil away from the criminalization of substance use to prevention and treatment, incarceration rates for even casual users remain high (Boiteux and Wiecko, 2009), a trend exacerbated under the Bolsonaro presidency. Despite this, there are several avenues that the individual can take to receive treatment. Most treatment is provided through the Unified Health System (SUS), which offers free health care to all Brazilian citizens, mainly through primary care clinics. With respect to mental health, within SUS there is a system of *Centros de Atenção Psicosocial* (CAPS) or Psychosocial Care Centers, and more specifically there are the CAPS-AD, or centers devoted to the treatment of alcohol and drug abuse. These community-based centers provide a continuum of multidisciplinary outpatient care, with the goal being a reduction in psychiatric hospitalization, including for drug abuse (Ferreira-Furegato et al., 2012).

Another major source of treatment for substance misuse are the *communidades terapeúticas* or therapeutic communities (CT). While CAPS-AD is purely outpatient, persons attending the CTs are required to live for several months in the community, usually tending gardens and small animals and participating in both group and private therapy sessions. In Brazil CTs are generally associated with religious organizations, and typically with evangelical or Pentecostal protestant churches (Lucchetti et al., 2016).

In Ribeirão Preto there is one CAPS-AD and several CTs. The CAPS-AD is somewhat unique in that, prior to the establishment of SUS, it was a mental health treatment center associated with the Spiritist movement in the state of São Paulo. Briefly, the Spiritist movement is associated with the writings of the 19th century figure Allan Kardec, who developed a belief system centered around the continuing moral evolution of the spirit after death, the ability of some to communicate with those spirits, and a commitment to social welfare (Greenfield, 2008). The CAPS-AD began as one such center and was the established in 1996 in accordance with changing legislation for mental health care in Brazil.

Of the several CTs in the city, we focused on two that were well outside the city center. The CTs consisted of fairly large *chácaras* (a term used in Portuguese to describe small farms or country houses), and patients live on-site for three to nine months of treatment. One CT was associated with the Catholic Church while the other was Pentecostal, and all residents were required to participate in religious study. Residents lived in dormitory-style buildings with several residents to a room and communal bathrooms. They were required to make their beds and clean the bathrooms, as well as work in the kitchens, tend the gardens, and take care of domestic animals. Opportunities for recreation included fishing in small ponds on the property and playing *fútebol* (soccer). During their stay, patients were allowed outside visitors only infrequently and under controlled conditions, the rationale being that separation encourages greater concentration on treatment. In addition to religious study, treatment consisted of group discussions as well as individual counseling sessions.

## Cultural models of substance use and stigma

An initial study of cultural models of substance use and attributed stigma was carried out sequentially in mid-2017 among a general population sample consisting of young adults (Henderson and Dressler, 2020). Participants were recruited through professors and students at two local universities and also at popular young adult hangout locations, such as a local shopping mall in Ribeirão Preto. It was reasoned that this age group was where the cultural model was socially “located” (i.e., most salient). There were several reasons to suspect this, including the fact that this group had most recently been the focus of drug education programs in secondary school while, at the same time, only beginning experimentation with substance use. Furthermore, they are high consumers of popular media that portray substance use and misuse. For these reasons, they serve as a kind of social repository of the schema that frame substance use and its evaluation.

A convenience sample of 16 young adults were asked to list factors associated with the risk of substance use, although data saturation (i.e., minimal generation of novel terms) was achieved with only 12 individuals. Twenty-nine items were retained for further analysis. Next, a second convenience sample of 35 respondents performed an unconstrained pile sort of these items. Multidimensional scaling and cluster analysis of the pile sort data indicated that the 29 risk factors were grouped into four categories: (1) social life (such as the influence of friends and going to parties or clubs); (2) the family (such as a family history of substance use and family problems); (3) self-medication (such as using drugs to seek relief, feeling anxious or depressed, wanting to feel better); and, (4) hedonism (having a lot of money, having a “weak head”). Additionally, 48 young adults rated each of the 29 risk factors on a 4-point scale from the risk factor having no influence on the risk of substance use to the risk factor being very influential (Henderson and Dressler, 2020).

Using cultural consensus analysis, the pile sort configuration was found to be highly shared; that is, there was strong agreement on the allocation of each risk factor to each of the four major categories. When the ratings of the influence of the risk factors were analyzed with cultural consensus analysis, however, there was no consensus. Further analysis with the internal consistency model for cultural consensus indicated that there was substantial agreement among respondents [respondent reliability = 0.871; see Weller (2007) for a discussion of different models for analyzing cultural consensus]. There were two reasons for the difference in these results between the cultural consensus model and the internal consistency model. First, the young adults tended to rate every risk factor as having potential influence on substance use, and cultural consensus analysis does not work well with these kinds of skewed ratings. Second, there was substantial residual agreement (Dressler et al., 2015) in the sample. One subgroup of respondents tended to rate psychosocial problems and self-medication as more important risk factors, while the other subgroup of respondents tended to rate social and hedonistic factors as more important.

What this means substantively is that, while there is an underlying cultural model of substance use risk, the model itself is not very specific. There is fairly high agreement on the elements of the model (risk factors) and their configuration; then, all the risk factors within that model are thought to be potential influences. In other words, nearly any path can lead to substance misuse.

A further finding of this preliminary study was the association between knowledge of the cultural model and the attribution of stigma to drug users. The residual agreement analysis was important in this respect: respondents who rated psychosocial problems and self-medication as more important also stigmatized drug users more, while respondents who rated social aspects of drug use and hedonism higher were less likely to stigmatize drug users (Henderson and Dressler, 2020). The “self-medicator” was deemed to be more untrustworthy and dangerous than the “feckless partier.”

With these results as a foundation, we initiated a study of persons under treatment for substance use. Of the factors likely to be influenced by cultural distance from the general population sample, here we focus on perceived stigma and self-stigma (Corrigan et al., 2017). Perceived stigma refers to the degree to which persons under treatment understand stigma to be prevalent in the society around them. Self-stigma, on the other hand, is the degree to which persons under treatment themselves stigmatize persons, including themselves, with substance use disorders. How do individuals under treatment view the risk of substance use relative to the general population? What are the implications of being proximate versus being distal from the general population sample in terms of perceptions of risk? We next turn to these questions.

## Materials and methods

Human subjects approval was received from the University of São Paulo-Ribeirão Preto (Approval No. 3.008.0012) and from The University of Alabama (Approval No. 17-OR-082-R1).

### Sampling

As noted above, research focused on individuals under treatment at the CAPS-AD (psychosocial treatment center for alcohol and drugs) in the community and in two of the CTs (therapeutic communities). Convenience sampling methods were utilized and all interviews were conducted in 2019. In the CAPS-AD, the lead author and a research assistant spent virtually every weekday in the clinic for close to nine months. All new patients were invited to participate in the research and made up about half of the sample from that clinic. Continuing patients made up the other half, who were interviewed when they attended the clinic for activities. Data were gathered in a semi-structured interview that lasted 60 to 90 min. Given that access to the CTs was much more limited, specific days (usually a Saturday) were designated for interviewing. This continued until all persons under treatment who agreed to participate in the research were interviewed. This resulted in a sample of 133 individuals.

Descriptively, the sample was predominately male (85%), although the CT samples skewed this due to the fact that they were male-only facilities. The sample was made up of adults (*m* = 38.14, s.d. = 11.85, range = 18–71), 62.4% of whom were single, 65.4% had children, and slightly over one-third (33.9%) had graduated from secondary school. Sixty-nine percent (*n* = 91) of the sample were drawn from CAPS-AD, while 31% (*n* = 41) were drawn from the CTs.

### Variable measurement

Interviews focused on the patient’s personal experience with the initiation and continuation of alcohol/drug use, their perceptions of the importance of the risk factors for substance use identified in the study of young adults in the general population, and structured scales to assess perceived social stigma and internalized self-stigma, the former referring to patient perceptions of stigma directed toward them and the latter referring to stigma directed inward.

With respect to the rating of the influence of risk factors, the young adults in the general public sample were primed to think about how community members broadly understood risk factors associated with addiction. In contrast, persons under treatment were specifically primed to report their *personal beliefs* regarding substance use risk. They rated each potential risk factor on a 4-point scale (1 = no influence; 2 = a little influence; 3 = some influence; 4 = a lot of influence) in terms of how that factor had affected their own personal drug use, or how they had seen that risk factor influence other substance users in their personal social network. These ratings capture individuals’ internalized beliefs about risk. When analyzed in terms of similarities and differences between the general population and treatment group samples, these data are used to plot the distribution of respondents in a space defined by cultural models of substance use risk. A cultural distance metric is then calculated using this array (see below).

A 14-item scale of perceived stigma was employed (Link et al., 2004). This scale, which had previously been translated into Portuguese, included items such as “Did any of your friends reject you after they found out about your alcohol or drug use?,” which was one of 6 dichotomous items, and “Most people believe that people who use drugs or alcohol cannot be trusted,” which was one of 8 items rated on a 4-point rating scale. This scale had acceptable reliability (alpha = 0.74).

Self-stigma was measured with an adaptation of Oliveira et al. (2015) Brazilian translation of the Internalized Stigma of Mental Illness Scale, which was originally designed to measure the experience of self-stigma broadly among persons with mental illness. For our use, “alcohol or drug use” was substituted for the term “mental illness.” Sample items include: “I am embarrassed or ashamed that I use alcohol or drugs;” “I feel inferior to other people because I use drugs or alcohol;” and, “Negative ideas or stereotypes about people who use drugs or alcohol apply to me.” This scale also had acceptable reliability in this sample (alpha = 0.89).

### Analysis and results

**Figure 1:**
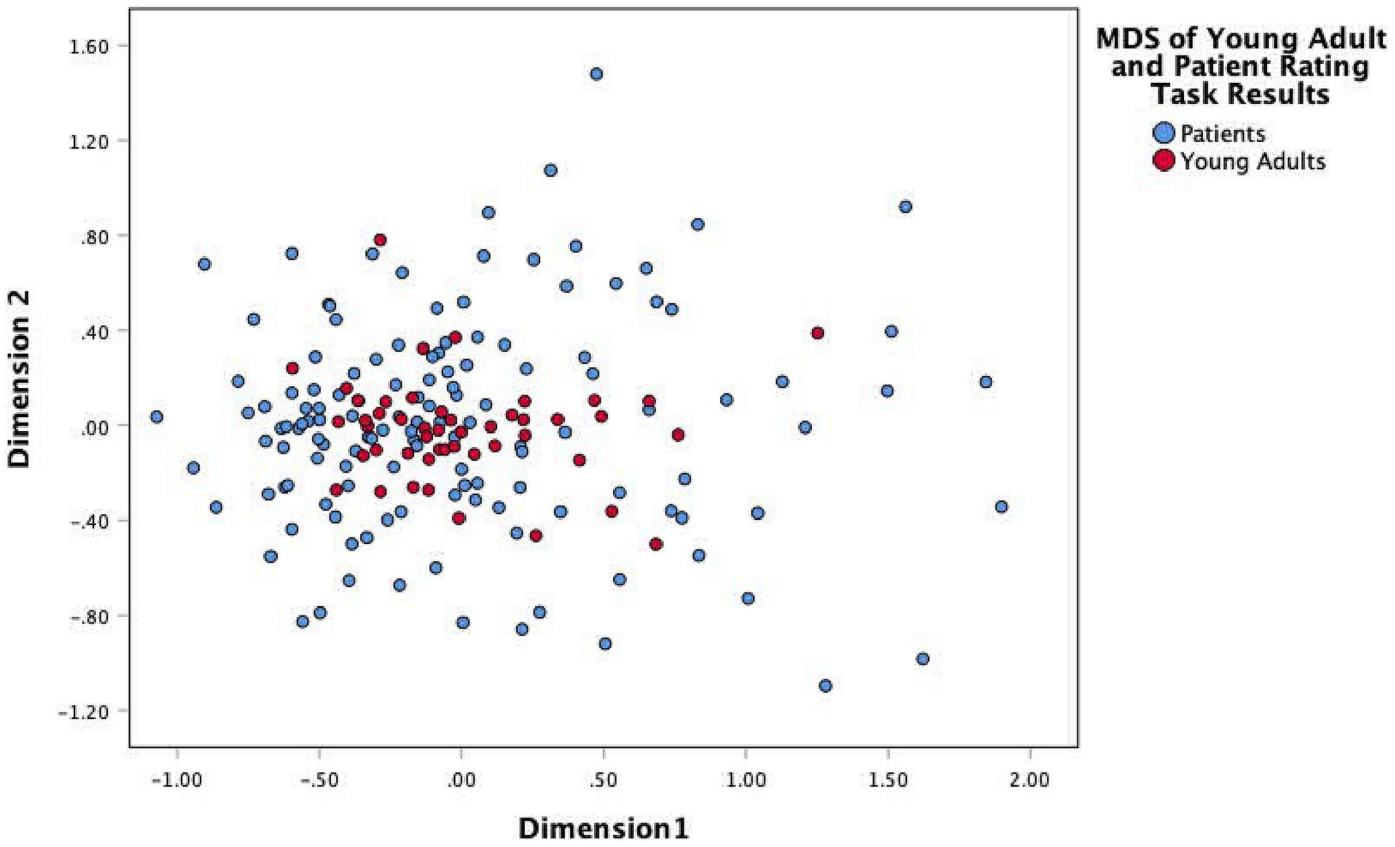
Nonmetric multidimensional scaling of distance of members of the treatment group (blue markers) from the general population sample (red markers).

Both Q-mode and R-mode analyses were employed with these data. First, a Q-mode (case-by-item) analysis was performed to measure and visualize the distance of individuals in the treatment group (henceforth TG) from the general population sample (henceforth GPS). Data were pooled for the two studies, with individuals as columns and the 29 ratings of the influence of risk factors on substance use as the rows. Then, following Garro (1986) and Chavez et al. (1995) nonmetric multidimensional scaling (MDS) was employed to scale a full symmetric matrix of profile dissimilarities. A two-dimensional solution (stress = 0.24) for this analysis is acceptable (see Sturrock and Rocha, 2000) and provides a visual representation of the distribution of cases (see Figure 1).

The members of the GPS are clustered toward the center of the graph, indicating both their relatively strong agreement on how the risk factors are configured and that all of the risk factors are influential with respect to substance use. The members of the TG, on the other hand, are widely distributed relative to the members of the GPS; some members of the TG are proximate to the GPS, while others are quite distant. This demonstrated that there were not two distinct cultural models for the two sample types as there was no indication that patients were moving toward the development of their own subcultural model. Rather, the young adults form a clear “cultural core” of the model, and the patients vary from this model in alternative ways. While there are differences in the way that members of the general public understand substance misuse risk, there are far more differences in terms of the ways that the patients come to internalize and believe in the influence of particular risk factors. In other words, the patients are not starting from scratch, but rather are beginning from the shared cultural model and using their personal experience to guide their shifts away from or toward the center of the cultural model.

At this point, the implications of the cultural distance of individuals in the TG from the GPS were explored to identify the extent to which the individual patient’s internalized beliefs differed from the “cultural core.” First, the distances of TG members from the GPS were calculated by subtracting the GPS centroid (or geometric center) from each individual TG member’s multidimensional scaling coordinates, then squaring and summing that difference. This provided a squared Euclidean distance metric for each TG member from the GPS as a whole, or:

$\begin{array}{c} \mathrm{Cultural}\;\mathrm{distance}={\left(TG_{\dim.1\;\mathrm{coord}.}-GPS_{\dim,1\;\mathrm{centroid}}\right)}^{2}\\ \;+\;{\left(TG_{\dim.2\;\mathrm{coord}.}-GPS_{\dim.2\;\mathrm{centroid}}\right)}^{2}\text{.} \end{array}$

Next, we turned to an R-mode (item-by-case) analysis of the TG ratings of risk factor influence. Like the GPS, the TG tended to rate most of the items as influential. Using exploratory factor analysis (varimax rotated principal components analysis), a 2-factor solution was obtained for the ratings. The 2-factor solution was selected primarily on the basis of a scree plot. Given the skewed values of the ratings, the correlations among the ratings of the risk factors were attenuated, resulting in a number of eigenvalues hovering around 1.0; however, the “elbow” in the scree plot clearly indicated a 2-factor solution, shown in Table 1. While the amount of variance explained by the two factors was modest (28%, again a result of the skewed ratings), the solution clearly indicates two distinct sets of risk factors as important from the perspective of the TG. The first factor combines risk factors that were distributed across all four of the risk factor clusters employed by the GPS. The dominant risk factors on Factor I include having a weak head, to rebel, easy access, influence of friends, emotional problems, going to parties and clubs, and desire for acceptance, as well as many others. Factor II includes only the items related to the basic sensations engendered by substance use. Keeping in mind that respondents in the TG rated items on the basis of their own experience and beliefs, we refer to Factor I as “Internalized Psychosocial Model of Risk” (IPSMR), and Factor II as “Internalized Experiential Model of Risk” (IEMR). The raw scores for each set of variables were summed to provide measures of each factor (and each has acceptable reliability, alpha = 0.84 and alpha = 0.68 respectively).

**Table 1:** Factor analysis of perceived influence of risk factors in the treatment group.

Risk factor	Factor 1	Factor 2
Weak head	**0.626**	0.055
To rebel	**0.578**	0.079
Easy access	**0.565**	0.017
Influence of friends	**0.538**	0.060
Emotional problems	**0.533**	0.336
Going to parties/clubs	**0.525**	0.025
Desire for acceptance	**0.511**	0.123
Depression	**0.499**	0.218
Financial problems	**0.491**	0.277
Family history of addiction	**0.487**	0.076
Curiosity	**0.482**	0.113
Lonely or isolated	**0.482**	0.370
Stress	**0.480**	0.302
Addictive properties of alcohol/drugs	**0.459**	0.000
Lack of family structure/dialog	**0.450**	0.072
Predisposition to addiction	**0.431**	0.177
Believe that have control over use	**0.406**	0.326
Friends that use alcohol/drugs	0.353	−0.019
Family problems	0.372	0.110
Environment	0.350	0.123
A lot of money	0.133	0.201
Search for relief	0.286	0.270
Lack of god	0.250	0.272
Lack of knowledge	0.224	0.365
To escape reality	0.265	**0.555**
To feel better	0.118	**0.575**
To relax	−0.215	**0.657**
Search for pleasure	−0.127	**0.690**
Good sensation	0.024	**0.708**

Factor loadings > 0.40 are in bold.

The cultural distance measures were highly skewed to the right, so a log transform was applied; descriptive statistics for all variables are shown in Table 2.

**Table 2:** Descriptive statistics for variables included in the analysis.

Variable	Total sample (*n* = 133)
IPSMR*	51.1 (± 9.8)
IEMR**	15.8 (± 3.6)
Self-stigma	40.9 (± 10.1)
Perceived stigma	19.1 (± 4.1)
Cultural distance	−0.55 (± 0.59)

^*^Internalized psychosocial model of risk.

^**^Internalized experiential model of risk.

Correlations of cultural distance with IPSMR, IEMR, perceived stigma, and internalized stigma were examined. Linear correlations of cultural distance with IPSMR (r = − 0.38, *p* < 0.001) and IEMR (r = 0.23, *p* < 0.01) were small to moderate and inverse, while these correlations with both measures of stigma were close to zero; however, when nonlinear associations were examined, the addition of both quadratic and cubic components to the correlations were statistically significant for all variables, with the exception of perceived stigma (*p* ≤ 0.03).

**Figure 2:**
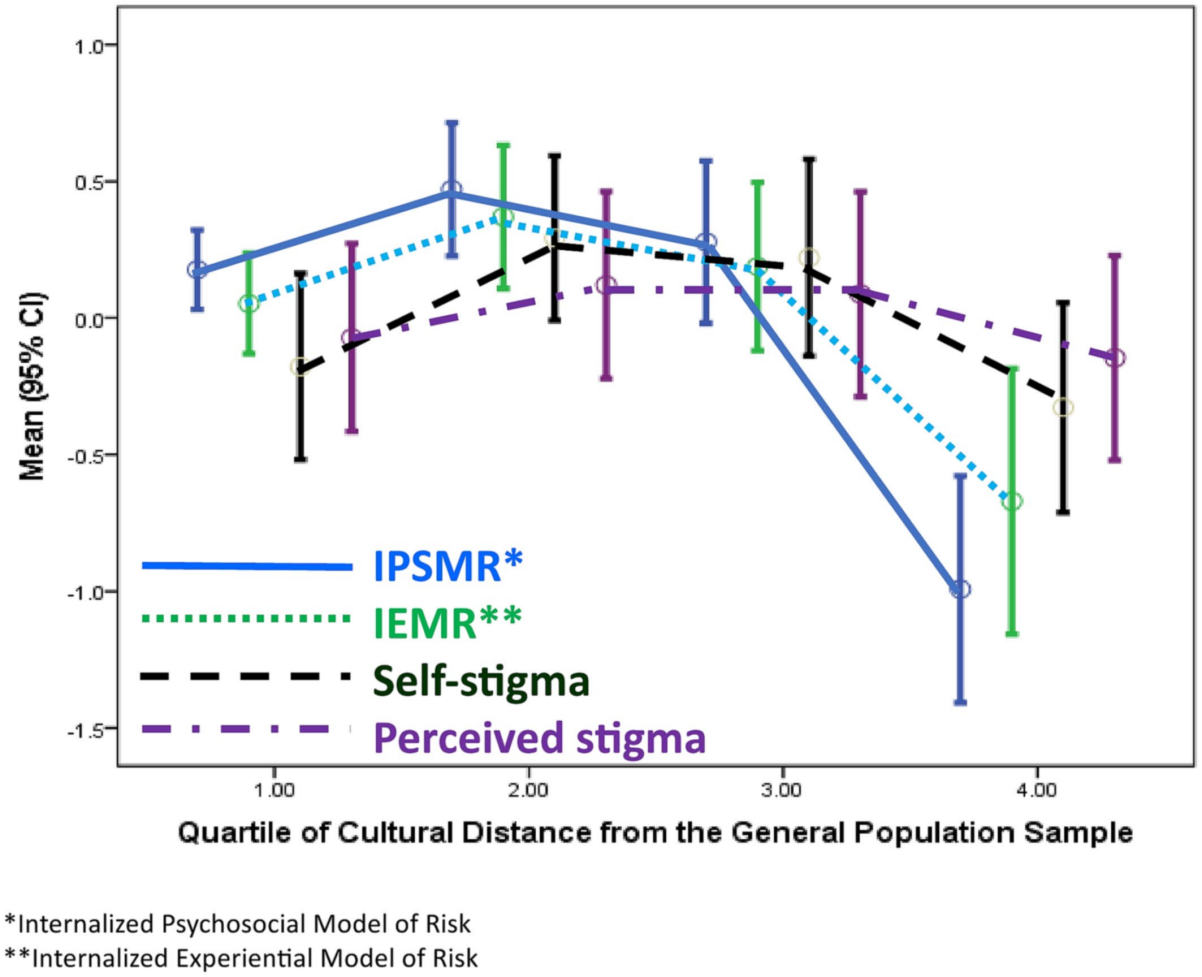
Association of internalized psychosocial model of risk, internalized experiential model of risk, self-stigma and perceived stigma with distance from the cultural model of substance misuse risk for members of the treatment group.

To display these associations more easily, cultural distance was divided into quartiles. Figure 2 shows the association of quartiles of cultural distance with the internalization scales and each of the stigma outcome variables; Table 3 shows means (± s.d.) for each scale by quartile of cultural distance, along with the analysis of variance for each scale. The associations of cultural distance quartile with IPSMR (eta = 0.583, *p* = 0.001) and IEMR (eta = 0.4045, *p* = 0.001) describe how the cultural model of substance misuse risk is reconfigured by distance from the GPS cultural model. While the members of the TG more proximate to the GPS have similar and high ratings of potential risk factors, the distal quartile reports significantly lower ratings of potential risk factors; furthermore, for both IPSMR and IEMR, the variance in the culturally distal quartile is higher than it is in any other group (*p* = 0.001).

**Table 3:** Means (± s.d.) of internalized psychosocial model of risk, internalized experiential model of risk, self-stigma, and perceived stigma by quartiles of cultural distance from the general population cultural model of substance misuse, with analysis of variance.

Quartile of distance from the general population cultural model	Internalized psychosocial model of risk	Internalized experiential model of risk	Self-stigma	Perceived stigma
1	52.8 (± 4.0)	15.9 (± 1.9)	39.1 (± 9.7)	18.8 (± 3.9)
2	55.0 (± 6.6)	17.0 (± 2.5)	43.9 (± 2.5)	19.6 (± 3.8)
3	54.2 (± 8.3)	16.5 (± 2.9)	43.2 (± 10.1)	19.5 (± 4.2)
4	41.4 (± 11.4)	13.4 (± 4.7)	37.6 (± 10.9)	18.5 (± 4.3)
Overall F-ratio (df = 3,129)	22.1**	8.4**	3.1*	0.6
F-ratio Linear Effect (df = 1,129)	33.1**	10.6**	0.5	0.7
F-ratio Nonlinear Effect (df = 2,129)	16.5**	7.3**	4.4*	0.4

*p < 0.05,

**p < 0.001.

Turning to the outcome variables, internalized stigma (eta = 0.262, *p* = 0.013) differs across the quartiles and deviates from linearity; the association of cultural distance quartile and perceived stigma is essentially zero (eta = 0.114, *p* = 0.461).

Post-hoc tests for IPSMR, IEMR, and internalized stigma indicate that the culturally distal quartile is significantly lower on those measures than the other three groups (*p* = 0.001).

## Discussion

In this study we examined how a group under treatment for substance use disorder in urban Brazil resembled a general population sample in their ratings of the influence of risk factors for substance use. Two forms of analysis were used. First, using a Q-mode, or case-by-variable analysis, we examined the profile similarity of each member of the treatment group to the general population sample in terms of the influence ratings. In this form of analysis, individual differences in the magnitude of the ratings of influence are ignored in favor of concentrating on patterns of similarity and difference. This analysis indicated substantial variation in the pattern of ratings among the members of the treatment group, relative to the general population sample. When visualized using nonmetric multidimensional scaling, this indicated that, while the general population sample was clustered together in their agreement on the potential influence of risk factors, the treatment group was widely scattered in terms of the profile similarity of their ratings to the general population sample. It is worth keeping in mind here that, in the general population sample, essentially all of the risk factors were considered to be at least a potential influence on the development of substance use disorder. The results of the Q-mode analysis show that there are distinct differences in this pattern among some of the members of the treatment group. From this analysis we derived our cultural distance metric, calculated as the distance of individual members of the treatment group from the center of the general population group configuration.

Second, using an R-mode, or variable-by-case analysis, we examined the differences in magnitude [or what Cronbach and Gleser (1953) originally described as “elevation” of scores] of ratings of the influence of risk factors relative to the distance of the members of the treatment group from the general population sample. This was done in terms of the two factors representing how the variables clustered for the treatment group, one factor composed of psychosocial risk factors, the other composed of the hedonic experience of drugs. In terms of both these factors, the further a member of the treatment group was from the general population sample, the lower they rated risk factors as influential in terms of substance misuse. This was especially true of the treatment group members most distal from the general population sample. Furthermore, within this distal group, the variability in ratings was significantly higher.

In our results we presented this in terms of mean values of the two factor scales. It is instructive, however, to look at this in a slightly different way. In a follow-up analysis, we dichotomized the ratings as influential (a rating of 3–4 on the Likert-response scale) versus not influential (ratings of 1–2 on the Likert-response scale), using all 29 items. The mean number of items rated as influential by quartile of distance from the general population sample was as follows: 22.2, 23.2, 20.6, and 14.5 (*p* < 0.001). As members of the treatment group diverge from the general population sample in the pattern of their ratings of influence, the actual number of items that they rate as influential drops from about 22 of 29 to about 14 of 29. In other words, the distal members of the treatment groups are making more distinctions among the potential risk factors, rather than viewing them as generally potent influences on the risk of substance misuse (It is worth noting, too, that members of the distal quartile of the treatment group who approach two standard deviations below the mean rating for that group are actually rating only 3–4 potential risk factors as influential).

The importance of distance from the general population sample in terms of beliefs about the influence of risk factors is further highlighted by the relationship with self-stigma. The members of the treatment group most distant from the general population cultural model are significantly less likely to stigmatize themselves and other substance users for their substance use, even though they are equally likely to perceive stigma against substance users as prevalent in the society around them.

As we noted earlier, unlike much research on cultural models that examines positively valued life goals or basic features of everyday life, we are examining here a cultural model of culturally constructed deviance. Substance use can be positively valued by some for its recreational, therapeutic, or spiritual value; substance misuse, however, is considered deviant. Chentsova-Dutton and Ryder (2020) suggested that cultural models theory could be profitably applied to the study of culturally constructed deviance. In their framework, they see this culturally constructed deviance as a kind of reflection of normalized cultural models, and for each kind of model the behavior that is culturally scripted can be valued, “unmarked” (by which they mean neither valued nor disvalued), or disvalued. They present this model as a 2 × 3 contingency table with normalized versus deviant models on the rows, and valued, unmarked, and disvalued presentations of those models in the columns. For example, with respect to drugs, psychoactive drugs can be valorized or simply normalized (i.e., unmarked) with respect to their use in orthodox medical practice to achieve the alleviation of common symptoms of anxiety or depression; on the other hand, even individuals who are under formal treatment for mental health disorders can be thought of as overly dependent on psychoactive drugs (i.e., the practice is disvalued).

With respect to the culturally scripted practices associated with substance misuse, while we did not examine this directly, the Chentsova-Dutton and Ryder (2020) model suggests that even deviant behavior can be valorized. In Brazil, heavy drinking and cocaine use are often associated with highly successful, wealthy, and powerful individuals, both because they can afford such expensive psychoactive recreation, and because as persons of higher social status they can avoid penalties in the criminal justice system for their behavior. Hence, while considered deviant, such individuals are ruefully granted social status.

More directly relevant to our results are Chentsova-Dutton’s and Ryder’s categories of unmarked and disvalued cultural scripts for substance misuse. What we found among the general population sample to be a prototype of the “feckless partier” is considered to be fairly common among college-age young adults, given that substance use is widely practiced at social events. While some people regard this as problematic, many simply shrug their shoulders and say that is just the way it is. This could represent the unmarked category in the Chentsova-Dutton/Ryder model. As we found, too, this prototype of substance use is not stigmatized in Brazil (Henderson and Dressler, 2020).

The cultural script for deviant and disvalued practices, then, is the “self-medicator:” the individual who seeks relief through substance use from the mental distress associated with social and family problems. And for the general population sample, this is the prototype of the substance user that is stigmatized (Henderson and Dressler, 2020).

The treatment group, however, seems less sanguine about the distinction between the partier and self-medicator in that in their configuration of risk factors both sets are combined as a single factor. What is more important with respect to alleviating their self-stigma is refining, and, we think, personalizing the inventory of risk factors. As this segment of the treatment group distances themselves from the cultural model of substance use in the general population, and as they narrow down the number of risk factors they regard as truly influential, they in turn suffer less self-stigma.

These results are consistent with Strauss’s conceptualization of the “subjectivities” of cultural models, and they complement, using a mixed-methods approach, her argument (Strauss, 2018a). While those members of the treatment group most proximate to the general population sample appear to simply take that cultural model as given (although they do aggregate risk factors in a way the general population does not), treatment group members who are distal from the general population appear to be reconfiguring the potential risk factors in novel ways, given their own experiences with substance use and, no doubt, other contextual factors. It is worth emphasizing here that the general population and the treatment group are operating with a common information pool of what constitutes risk factors. How they differ is in how they configure those risk factors, with the general population neatly compartmentalizing the factors into four groups (we think influenced strongly by their secondary school drug education), while the treatment group integrates the risk factors in novel ways (we think based on experience).

The results are consistent, too, with Spiro’s (1997) theory of internalization. The students and other young adults in the general population sample certainly know about substance misuse risk factors. The members of the treatment group, especially those who are distal from the general population sample, are using their combined cultural and personal models to understand the world and their lives in a particular way, which in turn is associated with their subjective well-being, in the sense of self-stigmatizing, or not.

We do not, however, have data on the process by which personal cultural models or “models in use” are constructed, although we suspect that Archer’s (2010) arguments regarding the importance of reflexivity are relevant here [see Caetano (2015) for a useful summary]. While reflexivity with respect to cultural models, meaning raising such models to full consciousness, has long been considered to be important for understanding culture and the individual, Archer has suggested that reflexivity can be considered a kind of individual difference variable, with individual variation in how persons achieve such a reflexive understanding of cultural models. For Archer, internal dialogue is an essential part of the process. This would suggest that individuals under treatment for substance misuse who are more distal from the general population may engage in an internal dialogue regarding substance misuse risk in which they are able ultimately to raise the general population model to consciousness and compare it to their own experience. Furthermore, Archer (2010) labels one mode of this internal dialogue as “communicative reflexivity,” suggesting that individuals who practice it seek confirmation of their thinking from others.

This is certainly consistent with activities in the CAPS-AD and therapeutic communities where treatment took place. Individual therapy was available, but group therapeutic groups were particularly important. The discussions in these groups could certainly be the locus of communicative reflexivity where individuals could share their experiences and receive confirmation of interpretations that both personalized those experiences and leavened the social stigma felt by the participants. This in turn would reinforce the perception of stigma in the larger society, while helping reduce the felt self-stigma. We are reminded of one of our respondents in the treatment group who, when asked to rate the influence of the risk factors, commented: “These are the sorts of things that people who do not abuse drugs think causes it.”

We collected some data on the treatment process and examined these in relation to cultural distance, perceived stigma, and self-stigma. The variables included: time in treatment, participation in treatment groups, participation in other activities, and individual treatment sessions with a psychologist. There was a weak tendency for persons participating in treatment groups and having individual sessions with the psychologist to have smaller cultural distance scores (i.e., to be closer to the general population model) and to report greater perceived stigma (*p* < 0.10). We suspect these are a function of being relatively new patients. A problem with these data is that they are simply self-reports of participation or not, with no indication of the actual degree of participation nor the quality of the interactions, and the self-reports of time spent in treatment are somewhat unreliable. Examination of the importance of reflexivity in this process would require carefully coded data regarding interaction and discourse in these treatment activities, and this should be examined more closely in future research.

These results are a further example of the utility of what Dressler et al. (2023) call a “spatial representation of culture.” In this conceptualization, we as individuals are seen as inhabiting a cultural space, defined by the parameters of the cultural model for any specific domain. In an analysis of culturally constructed adult developmental life goals, Dressler et al. found that individuals who were distant from the prototype of one achieving those life goals reported higher psychological distress, due to their perceived (by self and others) difficulty in navigating that social space.

The results presented here examine the other side of achieving normalcy, in Chentsova-Dutton and Ryder’s (2020) sense. The prototypes for substance misuse are the partier and the self-medicator, with the latter stigmatized in the general population cultural model. The more that the substance user under treatment can distance themselves from this prototype, the less they engage in self-stigma. In this case, being culturally marginalized appears to ameliorate the distress they experience. Conceptualizing this in spatial terms is thus useful.

It is worth noting, too, that this analysis and the measurement of cultural distance are based on an emic approach; that is, the terms that make up the cultural model of risk factors for substance misuse were elicited from members of the community and knowledge of these risk factors was shown to be shared both in the general population sample and the treatment group. The measure of cultural distance between members of the treatment group and the general population sample can thus be said to have high “emic validity” (Dressler and Oths, 2014) in that it locates individuals along a continuum defined in the terms that they themselves use to talk about substance use. This emic validity thus lends credence to the findings.

There are of course limitations to this study. First, the sample from which the data were collected is a convenience sample and individuals self-selected into the study. Testing hypotheses derived from our study with a sample of persons under treatment for substance misuse that better reflects the larger population of persons under treatment would be useful. Second, it is also noteworthy that model construction among the general public occurred in mid-2017, while interviews and data collection with patients occurred throughout 2019. Although the authors do not have reason to believe that understandings of substance use/misuse shifted significantly during this time, they may have. Third, as noted above, the measure of cultural distance of persons from the cultural model of risk as defined by the general population has high emic validity, based as it is on a careful cultural domain analysis carried out in this particular community. This raises the question, however, of how widely this cultural model might be distributed. Brazil is a heterogeneous society with distinct regional differences in history and society that might influence how cultural models of substance use/misuse are configured. Future research on this question would also be useful.

This line of inquiry can be extended in future research to understand better how persons who are considered marginal and are stigmatized use cultural models that are imposed upon them to reconstruct personal models supporting, we hope, their well-being.

## Data availability statement

The raw data supporting the conclusions of this article will be made available by the authors, without undue reservation.

## Ethics statement

This study was carried out with the recommendations of the Institutional Review Board for the Protection of Human Subjects of The University of Alabama and the Ethics Committee of the Faculty of Nursing of the University of São Paulo-Ribeirão Preto. All subjects gave written consent in accordance with the Declaration of Helsinki. The studies involving humans were approved by Institutional Review Board for the Protection of Human Subjects, The University of Alabama, Tuscaloosa, Alabama, USA Committee for Ethics in Research, Faculty of Nursing, University of São Paulo-Ribeirao Preto, Brazil. The studies were conducted in accordance with the local legislation and institutional requirements. The participants provided their written informed consent to participate in this study.

## Author contributions

NH: Conceptualization, Formal analysis, Methodology, Writing – original draft, Writing – review & editing, Data curation, Funding acquisition, Investigation. WD: Writing – review & editing, Conceptualization, Formal analysis, Methodology, Writing – original draft. NP: Writing – review & editing, Project administration, Supervision. AF: Investigation, Writing – review & editing. SP: Investigation, Project administration, Supervision, Writing – review & editing.
